# Evaluating the Contributions of Individual Variables to a Quadratic Form

**DOI:** 10.1111/anzs.12144

**Published:** 2016-03-21

**Authors:** Paul H. Garthwaite, Inge Koch

**Affiliations:** ^1^ Department of Mathematics and StatisticsThe Open UniversityMK7 6AAUK; ^2^ School of Mathematical SciencesUniversity of AdelaideSA 5005Australia

**Keywords:** Corr‐max transformation, collinearity, discriminant analysis, Hotelling, Mahalanobis distance, rotation

## Abstract

Quadratic forms capture multivariate information in a single number, making them useful, for example, in hypothesis testing. When a quadratic form is large and hence interesting, it might be informative to partition the quadratic form into contributions of individual variables. In this paper it is argued that meaningful partitions can be formed, though the precise partition that is determined will depend on the criterion used to select it. An intuitively reasonable criterion is proposed and the partition to which it leads is determined. The partition is based on a transformation that maximises the sum of the correlations between individual variables and the variables to which they transform under a constraint. Properties of the partition, including optimality properties, are examined. The contributions of individual variables to a quadratic form are less clear‐cut when variables are collinear, and forming new variables through rotation can lead to greater transparency. The transformation is adapted so that it has an invariance property under such rotation, whereby the assessed contributions are unchanged for variables that the rotation does not affect directly. Application of the partition to Hotelling's one‐ and two‐sample test statistics, Mahalanobis distance and discriminant analysis is described and illustrated through examples. It is shown that bootstrap confidence intervals for the contributions of individual variables to a partition are readily obtained.

## 1 Introduction

Quadratic forms feature as statistics in various multivariate contexts. Well‐known examples include Hotelling's $T^{2}$ statistic and the Mahalanobis distance. When the value of a quadratic form is large, then an obvious question is: *Which variables cause it to be large?* To illustrate, suppose **x** is an observation that should come from a distribution with mean ***μ*** and variance **Σ**. However, it appears to come from a different distribution because the Mahalonobis distance, equal to the quadratic form ${\left(x-\mu \right)}^{⊤}\Sigma^{-1}\left(x-\mu \right)$, is large. It might be helpful to have a measure of the contribution of individual variables to the size of this quadratic form.

When variables are correlated, it is not immediately apparent that a sensible answer to this question can be given. However, we shall argue that the question can be answered in a meaningful way and we will propose a method of partitioning a quadratic form into contributions from individual variables. This does not imply that there is a “best” way of forming such a partition, other than in some simple situations where arguments of symmetry can be used. However, although a partition of a quadratic form may be arbitrary to a degree, it can still be useful and informative. We show that the partition we propose meets certain optimality criteria.

Our method of forming a partition is based on a transformation that we call the corr‐max transformation. Garthwaite, Critchley, Anaya‐Izquierdo & Mubwandarikwa (2012) focussed on a transformation referred to as the cos‐square transformation, but also proposed a second transformation called the cos‐max transformation. The latter is closely related to the corr‐max transformation that we introduce here. However, while the cos‐max transformation was designed to transform a data matrix, the intended use of the corr‐max transformation is the transformation of a random vector. The cos‐max transformation adjusts a data matrix by a minimal amount while yielding a matrix with orthonormal columns; each of the original variables is associated with exactly one of these columns. The corr‐max transformation yields a vector whose covariance matrix is proportional to the identity matrix, while each of the original variables is associated with exactly one component of the transformed vector. The strength of the associations is measured by correlations and the transformation is chosen to maximise the sum of these correlations (hence our name for the transformation).

Collinearities between variables will reduce the strength of some associations. The variables that are involved in a collinearity can be identified using the cos‐max transformation (Garthwaite *et al*. 2012). The coordinate axes corresponding to these variables can then be rotated to yield a set of variables with little collinearity. We adapt the corr‐max transformation so that contributions to the quadratic form, as measured by the partition, will only change for those variables that are affected by the rotation. We refer to this feature as the rotation invariance property.

The task of determining which variables have most influence on a Mahalanobis distance has attracted some attention in the literature. The Mahalanobis‐Taguchi system, which features in statistical process control, estimates the covariance matrix **Σ** from ‘normal’ data and computes the Mahalanobis distances for a set of ‘abnormal’ data points, in order to determine signal‐to‐noise ratios for individual variables and hence identify variables that are useful diagnostics of abnormality (Taguchi & Jugulum 2002; Das & Datta 2007). In ecology, the Mahalanobis distance has been used in the construction of maps that show suitable habitat areas for a particular species. Pixels on the map are equated to points in multidimensional space on the basis of environmental variables and the Mahalanobis distance is used to give a measure of the distance from a point to the mean of the ecological niche. To identify the minimum set of basic habitat requirements for a species, Rotenberry, Preston & Knick (2006) proposed a decomposition of the Mahalanobis distance that exploits the eigenvectors of **Σ**. Based on work in Rotenberry, Knick & Dunn (2002), they argued that the variables that load heavily on the eigenvectors corresponding to the smallest eigenvalues are the most influential in determining habitat suitability. Calenge, Darmon, Basille & Jullien (2008) added a step to the method of Rotenberry *et al*. (2006), forming a further eigenvector decomposition with the aim of producing biologically meaningful axes. The decomposition we propose here is simpler to implement and has a straightforward interpretation, making it more likely to be put into practice. Rogers (2015) adopted it as a tool for identifying the key climate variables in determining future changes in the distribution of vector‐borne diseases, illustrating its use through application to dengue, an important tropical disease. He referred to the contributions of variables being measured on the *Garthwaite–Koch scale*, citing a technical report (Garthwaite & Koch 2013) that forms the basis of the present paper.

In Section 2 we argue that the value of a quadratic form can be meaningfully partitioned into separate contributions of individual variables and give the criteria that determine the corr‐max transformation and our proposed partition. In Section 3 we obtain the transformation and the partition. In Section 4 the transformation is adapted to have the rotation invariance property and ways to exploit this property are suggested. In Section 5 we describe use of the partition in contexts where Hotelling's $T^{2}$ statistic or Mahalanobis distance arise, and in discriminant problems involving two groups. Bootstrap confidence intervals for the contributions of individual variables can be constructed to quantify uncertainty in these contributions and to increase our insight into the relative importance of these contributions. These ideas are illustrated in Section 6. Concluding comments are given in Section 7.

## 2 Rationale for a partition

Let *Q* be the quadratic form

(1) $$Q={\left(X-\mu \right)}^{⊤}\Sigma^{-1}\left(X-\mu \right),$$

where $X={\left(X_{1},\ldots ,X_{m}\right)}^{⊤}$ is an *m*×1 random vector whose variance is proportional to **Σ** and where ***μ*** is a given *m*×1 vector that is not necessarily the mean of ***X***. This type of quadratic form arises in various applications. For example, in Hotelling's one‐sample $T^{2}$ statistic, ***X*** would take the value of a sample mean, **Σ** would be the population variance, and ***μ*** would be the hypothesised population mean. The purpose of this paper is to give a method of evaluating the contributions of individual variables to *Q*. Before doing so, we must first consider whether it is possible, in principle, to meaningfully answer the question, *What are the contributions of individual variables to a quadratic form?*

Clearly a good answer can easily be given when **Σ** is the identity matrix: the contribution of each variable is then the square of the corresponding component of **x**−***μ***. Extension to the case where **Σ** is diagonal is obvious. However, if **Σ** is not diagonal then it is less clear that *Q* can be partitioned between variables in a meaningful way. To examine this issue, we consider an example.

Specifically, let

$$\mu =\left(\begin{array}{c} 0\\ 0\\ 0 \end{array}\right)\;\text{and}\;\Sigma^{-1}=\left(\begin{array}{ccc} 1 & 0 & 0\\ 0 & 1 & 0.3\\ 0 & 0.3 & 1 \end{array}\right)\;\;,$$

and, to aid explanation, suppose the three components of $x={\left(x_{1},x_{2},x_{3}\right)}^{⊤}$ correspond to standardised variables, *age* ($x_{1}$), *height* ($x_{2}$), and *weight* ($x_{3}$). In this example the contribution of *age* ($x_{1}$) to *Q* is always clear, since

$$Q=\left(x_{1},x_{2},x_{3}\right)\left(\begin{array}{ccc} 1 & 0 & 0\\ 0 & 1 & 0.3\\ 0 & 0.3 & 1 \end{array}\right)\left(\begin{array}{c} x_{1}\\ x_{2}\\ x_{3} \end{array}\right)=x_{1}^{2}+\left(x_{2},x_{3}\right)\left(\begin{array}{cc} 1 & 0.3\\ 0.3 & 1 \end{array}\right)\left(\begin{array}{c} x_{2}\\ x_{3} \end{array}\right)\;\;.$$

If $x_{2}=x_{3}$, then *height* and *weight* contribute equally to *Q*, from symmetry. Hence, even though $\Sigma^{-1}$ is not diagonal, the contributions of each variable to *Q* can be determined: *age* contributes $x_{1}^{2}$ while *height* and *weight* each contribute $(Q-x_{1}^{2})/2$.

To expand this example, suppose $x_{3}$ to be slightly greater in magnitude than $x_{2}$. Then the contribution of *age* to *Q* would still be $x_{1}^{2}$ while, in dividing the balance of *Q* between *height* and *weight*, it seems reasonable to give *weight* slightly the greater portion. Other situations are also readily constructed where common sense can indicate, approximately, the contributions of each variable to *Q*. Usually though, there will be no partition of *Q* that is unquestionably better than any alternative. However, it may still be the case that sensible methods of partitioning *Q* broadly agree on the contributions made by individual variables. We construct a partition that helps interpret the results of some statistical analyses by giving a clearer relationship between the data variables and a test statistic or some other quantity that is based on *Q*. The transformation that underlies the partition is defined in the next section.

Before ending this section we introduce further notation that will be used in the remainder of the paper. Bold‐face italic capital letters ***X***,***Y***, $W^{⋄}$, $\hat{W}$, etc. are *m*×1 random vectors. Subscripts are added to denote components of the vector: $X={\left(X_{1},\ldots ,X_{m}\right)}^{⊤}$, $\hat{W}={\left({\hat{W}}_{1},\ldots ,{\hat{W}}_{m}\right)}^{⊤}$, etc. The notation $\hat{\Sigma }$ is used to denote a generic estimate of the *m*×*m* population variance matrix, **Σ**. Likewise ${\hat{\Sigma }}_{1}$ is used to denote the standard unbiased estimate of **Σ** given by one sample and ${\hat{\Sigma }}_{p}$ is used to denote the standard pooled estimate of **Σ** based on independent samples from two populations that both have variance **Σ**. The symbols $\sigma_{i}^{2}$, ${\hat{\sigma }}_{i}^{2}$ are used to denote the *i*th diagonal entries of **Σ** and $\hat{\Sigma }$, respectively. The symbols **D** and $\hat{D}$ are used to denote *m*×*m* diagonal matrices with *i*th diagonal entries equal to $\sigma_{i}^{-1}$and ${\hat{\sigma }}_{i}^{-1}$, respectively (*i*=1,…,*m*). Thus **DΣD** and $\hat{D}\hat{\Sigma }\hat{D}$ have diagonal entries equal to 1. The symbol $X={\left(x_{1},\ldots ,x_{n}\right)}^{⊤}$ is used to denote the *n*×*m* data matrix whose rows are the *n* observations and ${\tilde{x}}_{i}$ is used to denote the *i*th column of **X**. The symbols **A**, $\hat{A}$, **B**,** C**,** H**,** Ω**,** Ψ** are used to denote *m*×*m* matrices and **Γ** and $\Gamma_{d}$ are used to denote *m*×*m* and *d*×*d* orthogonal matrices, respectively.

## 3 The corr‐max transformation

To form our partition, we consider transformations of the form

(2) X↦W=A(X−μ),

where ***W*** is an *m*×1 vector and

(3) $$W^{⊤}W={\left(X-\mu \right)}^{⊤}\Sigma^{-1}\left(X-\mu \right)$$

for any value of ***X***. Then

(4) $$Q=\sum_{i=1}^{m}W_{i}^{2},$$

where $W={\left(W_{1},\ldots ,W_{m}\right)}^{⊤}$, so ***W*** yields a partition of *Q*. The partition will be useful and meaningful if

- the components of ***W*** are uncorrelated and have identical variances, and
- it is reasonable to identify $W_{i}$ with the *i*th *x*‐variable, as the contribution of that *x*‐variable to *Q* can then sensibly be defined as $W_{i}^{2}$.

The following theorem gives the transformation that maximises $\Sigma_{i=1}^{m}\text{cor}\left(X_{i},W_{i}\right)$ under the constraints that (2) and (3) hold, where cor(·,·) denotes correlation. Proofs of theorems are given in Appendix A.

Theorem 1 Suppose ***W***=**A**(***X***−***μ***) and var(***X***)∝**Σ**. If (3) holds for all ***X***, then the components of ***W*** are uncorrelated with identical variances. If, in addition, **A** is chosen to maximise $\Sigma_{i=1}^{m}\text{cor}\left(X_{i},W_{i}\right)$, then $A={\left(D\Sigma D\right)}^{-1/2}D$, where **D** is a diagonal matrix such that **DΣD** has diagonal entries equal to 1.

We define the *corr‐max transformation* to be the transformation given by (2) with $A={\left(D\Sigma D\right)}^{-1/2}D$. From Theorem 1, this transformation yields a ***W*** that satisfies requirement (a). For (b), we first note that it is always possible to scale and translate $X_{i}$ so that it has the same variance and the same mean as $W_{i}$, whence the degree to which $X_{i}$ equates to $W_{i}$ would primarily be determined by its correlation with $W_{i}$. (Perfect correlation would imply that they were identical.) Moreover, scaling and translation do not change the nature of a variable. Otherwise, for example, temperature measurements on the Celsius and Fahrenheit scales would not be equivalent. Hence, the degree to which $W_{i}$ equates to the *i*th *x*‐variable is largely determined by cor$\left(X_{i},W_{i}\right)$. Consequently under a sensible criterion the corr‐max transformation satisfies (b) as fully as possible, since it maximises $\Sigma_{i=1}^{m}\text{cor}\left(X_{i},W_{i}\right)$ when the constraint equations (2) and (3) hold. The extent to which the corr‐max transformation satisfies (b) is discussed further in Section 7.

Theorem 1 completes the specification of our partition for the case where **Σ** is known. To summarise, if ***X*** takes the value **x** and var(***X***)∝**Σ**, the corr‐max transformation yields the new vector $w={\left(D\Sigma D\right)}^{-1/2}D\left(x-\mu \right)$ and the contribution of the *i*th *x*‐variable to $Q\left(x\right)={\left(x-\mu \right)}^{⊤}\Sigma^{-1}{\left(x-\mu \right)}^{⊤}$ is defined to be $w_{i}^{2}$ (*i*=1,…,*m*).

When **Σ** is unknown, we replace it in the foregoing method with an estimate, $\hat{\Sigma }$ say. In some contexts this type of substitution can have drawbacks but here it seems appropriate, since it yields properties similar to Theorem 1, but in terms of maximising sample correlations, which we denote by ${\text{cor}}_{s}\left(\cdot ,\cdot \right)$, rather than population correlations. This result is given in Theorem 2. Its proof is similar to that of Theorem 1 and is omitted.

Theorem 2 Suppose that the sample variance of ***X*** is proportional to $\hat{\Sigma }$ and $\Sigma_{j=1}^{m}{\text{cor}}_{s}\left(X_{j},{\hat{W}}_{j}\right)$ is to be maximised, subject to $\hat{W}=\hat{A}\left(X-\mu \right)$ and ${\hat{W}}^{⊤}\hat{W}={\left(X-\mu \right)}^{⊤}{\hat{\Sigma }}^{-1}{\left(X-\mu \right)}^{⊤}$ for any ***X***. Then $\hat{A}={\left(\hat{D}\hat{\Sigma }\hat{D}\right)}^{-1/2}\hat{D}$ and

(5) $$\hat{W}={\left(\hat{D}\hat{\Sigma }\hat{D}\right)}^{-1/2}\hat{D}\left(X-\mu \right),$$

where $\hat{D}$ is diagonal and $\hat{D}\hat{\Sigma }\hat{D}$ has diagonal entries equal to 1.

While the corr‐max transformation yields a sensible method of partitioning *Q* into contributions of individual variables, other reasonable methods may well give a slightly different partition, but differences should be small when there is a close relationship between each $W_{i}$ variable and the *x*‐variable with which it is paired. Information about the strength of these relationships is provided by the correlations between $X_{i}$ and $W_{i}$ (*i*=1,…,*m*). The following theorem gives a simple means of finding the values of these correlations and, more generally, the correlations $\text{cor}(X_{i},W_{j})$ and ${\text{cor}}_{s}\left(X_{i},{\hat{W}}_{j}\right)$ for *i*=1,…,*m*;*j*=1,…,*m*. It has the interesting implications that $\text{cor}\left(X_{i},W_{j}\right)=\text{cor}\left(X_{j},W_{i}\right)$ and ${\text{cor}}_{s}\left(X_{i},{\hat{W}}_{j}\right)={\text{cor}}_{s}\left(X_{j},{\hat{W}}_{i}\right)$ for all *i* and *j*, since ${\left(D\Sigma D\right)}^{1/2}$ and ${\left(\hat{D}\hat{\Sigma }\hat{D}\right)}^{1/2}$ are both symmetric matrices.

Theorem 3 Suppose $W={\left(D\Sigma D\right)}^{-1/2}D\left(X-\mu \right)$ and var(***X***)∝**Σ**. Then $\text{cor}(X_{i},W_{j})$ equals the (*i*,*j*)*th* entry of ${\left(D\Sigma D\right)}^{1/2}$. Similarly, if $\hat{W}={\left(\hat{D}\hat{\Sigma }\hat{D}\right)}^{-1/2}\hat{D}\left(X-\mu \right)$ and the sample variance of ***X*** is proportional to $\hat{\Sigma }$, then ${\text{cor}}_{s}\left(X_{i},{\hat{W}}_{j}\right)$ equals the (*i*,*j*)*th* entry of ${\left(\hat{D}\hat{\Sigma }\hat{D}\right)}^{1/2}$.

So far we have only considered the partition of a quadratic form, but the corr‐max transformation also gives a useful partition of the bilinear form $U^{⊤}\Sigma^{-1}V$, provided var(**U**)∝**Σ** and var(**V**)∝**Σ**. Theorem 4 gives the relevant result. Its proof follows from the proof of Theorem 1.

Theorem 4 Suppose var(***U***)∝**Σ** and var(***V***)∝**Σ** where ***U*** and ***V*** are *m*×1 random vectors. Let $W^{*}=AU$ and $W^{\circ }=AV$ where **A** is a square matrix. Under the constraint ${\left(W^{*}\right)}^{⊤}W^{\circ }=U^{⊤}\Sigma^{-1}V$, both $\Sigma_{i=1}^{m}\text{cor}\left(U_{i},W_{i}^{*}\right)$ and $\Sigma_{i=1}^{m}\text{cor}\left(V_{i},W_{i}^{\circ }\right)$ are maximised when $A={\left(D\Sigma D\right)}^{-1/2}D$.

Both $U\mapsto W^{*}$ and $V\mapsto W^{\circ }$ are corr‐max transformations, since $A={\left(D\Sigma D\right)}^{-1/2}D$. From this, and from the theorem, it is reasonable to identify the *i*th components of $W^{*}$ and $W^{\circ }$ with the *i*th components of ***U*** and ***V***, respectively. Then ${\left(W^{*}\right)}^{⊤}W^{\circ }$ is our partition of $U^{⊤}\Sigma^{-1}V$, giving $W_{i}^{*}W_{i}^{\circ }$ as the contribution of the *i*th *x*‐variable to $U^{⊤}\Sigma^{-1}V$. In Section 5 we use the theorem to form a partition of Fisher's linear discriminant function. When **Σ** is estimated from data, results corresponding to Theorem 4 hold with $\hat{A}={\left(\hat{D}\hat{\Sigma }\hat{D}\right)}^{-1/2}\hat{D}$.

## 4 Rotation invariance property

When the correlations between $X_{i}$ and ${\hat{W}}_{i}$ are weak for some values of *i*, there will generally be strong collinearities between some of the *x*‐variables. The standard diagnostic for detecting collinearities are variance inflation factors. Suppose the values of $X_{1},\ldots ,X_{m}$ are observed on each of *n* items (*n*>*m*) and let $R_{j}^{2}$ denote the multiple correlation coefficient when $X_{j}$ is regressed on the other *X* variables. Then the variance inflation factor for $X_{j}$, ${VIF}_{j}$ say, is defined to be ${\left(1-R_{j}^{2}\right)}^{-1}$. This will be large if $X_{j}$ is involved in a collinearity. Garthwaite *et al*. (2012) showed that the *x*‐variables involved in a collinearity can be identified using the cos‐max transformation. Example 2 in Section 5.2 illustrates this.

Collinear variables can be replaced by non‐collinear variables via orthogonal rotation of coordinate axes. This can clarify the relationship between *x*‐variables and a quadratic form, as examples will illustrate. Only axes corresponding to collinear variables need be rotated. The results of a rotation are sensitive to scale, so before rotation we scale the *x*‐variables. This is the same as in principal component analysis, where variables are frequently scaled to have identical variances before applying the principal component transformation (which is an orthogonal rotation).

Here var(***X***)∝**Σ** and **DΣD** has diagonal entries all equal to 1, so the components of **D** ***X*** have identical variances. Let **Γ** be an *m*×*m* orthogonal matrix and put ***Y***=**ΓD**(***X***−***μ***), so that ***Y*** is obtained by a re‐scaling of ***X***−***μ***, followed by an orthogonal rotation. Suppose that we want to apply a transform

(6) $$Y\mapsto W^{⋄}=CY,$$

in such a manner that there are large correlations between $Y_{i}$ and $W_{i}^{⋄}$ for *i*=1,…,*m*. The components of **Y** are not all equally important – after rotation some components will have a smaller variance than others and those with smaller variances are deemed to be less important, as in principal components analysis. The corr‐max transformation would choose **C** to maximise $\Sigma_{i=1}^{m}\text{cor}\left(Y_{i},W_{i}^{⋄}\right)$ but now, to reflect the differing importance of some variables, we choose **C** to maximise $\Sigma_{i=1}^{m}{\left\{\text{var}(Y_{i})\right\}}^{1/2}\text{cor}\left(Y_{i},W_{i}^{⋄}\right)$. This gives greater weight to the $Y_{i}$ with greater variance. Theorem 5 gives the resulting matrix **C** and properties of the transformation.

Theorem 5 Let ***Y***=**ΓD**(***X***−***μ***) where **Γ** is a given orthogonal matrix and var(***X***)∝**Σ**. Suppose $\Sigma_{i=1}^{m}{\left\{\text{var}(Y_{i})\right\}}^{1/2}\text{cor}\left(Y_{i},W_{i}^{⋄}\right)\}$ is to be maximised, subject to $W^{⋄}=CY$ and ${\left(W^{⋄}\right)}^{⊤}W^{⋄}={\left(X-\mu \right)}^{⊤}\Sigma^{-1}\left(X-\mu \right)$, where **C** is a square matrix. Then:

- the components of $W^{⋄}$ are uncorrelated and have identical variances;
- $$C={\left(\Gamma D\Sigma D\Gamma^{⊤}\right)}^{-1/2}=\Gamma {\left(D\Sigma D\right)}^{-1/2}\Gamma^{⊤};$$
- (7) $$W^{⋄}=\Gamma {\left(D\Sigma D\right)}^{-1/2}D\left(X-\mu \right);$$
- $\left[{\left\{\text{var}(Y_{i})\right\}}^{1/2}\text{cor}\left(\right.Y_{i},W_{j}^{⋄}\right]$ is equal to the (i, j)th entry of ${\left(\Gamma D\Sigma D\Gamma^{⊤}\right)}^{1/2}$;
- with $W^{⋄}$ written as $W^{⋄}\left(\Gamma \right)$ to highlight that it is a function of **Γ**,
  (8) $$W^{⋄}\left(\Gamma \right)=\Gamma W^{⋄}\left(I\right)$$
  where $W^{⋄}\left(I\right)=I{\left(D\Sigma D\right)}^{-1/2}D\left(X-\mu \right)$.

The transformation from ***X***−***μ*** to $W^{⋄}$ will be referred to as the *adapted* corr‐max transformation. It is identical to the ordinary corr‐max transformation if there is no rotation, that is when **Γ**=**I**. If **Σ** is unknown, we replace it with an estimate, $\hat{\Sigma }$, and put

(9) $${\hat{W}}^{⋄}=\Gamma {\left(\hat{D}\hat{\Sigma }\hat{D}\right)}^{-1/2}\hat{D}\left(X-\mu \right).$$

The contribution of the *i*th variable to the quadratic form is evaluated as ${\left(w_{i}^{⋄}\right)}^{2}$, where $w_{i}^{⋄}$ is the value taken by the *i*th component of $W^{⋄}$ or ${\hat{W}}^{⋄}$.

From equation (8) we obtain the same result whether (a) we multiply **D**(***X***−***μ***) by the rotation matrix **Γ** and transform the result, or (b) we transform **D**(***X***−***μ***) and multiply the result by **Γ**. That is, with the adapted corr‐max transformation, the operations of rotation and transformation are commutative.

This property allows us to rotate the coordinate axes corresponding to *x*‐variables involved in a collinearity while neither affecting the identity of other *x*‐variables, nor altering assessments of the latter variables’ contributions to the quadratic form. To elucidate, suppose that we want to rotate the first *d* of the *m* axes. Then the rotation matrix **Γ** has the following block‐diagonal form:

(10) $$\Gamma =\left[\begin{array}{cc} \Gamma_{d} & 0\\ 0 & I_{m-d} \end{array}\right]\;\;,$$

where $\Gamma_{d}$ is a *d*×*d* orthogonal matrix. Multiplying ***X*** by **Γ** only changes the first *d* components of ***X*** and leaves its other components unchanged, so the latter components are the original variables. Moreover, under the transformation in equation (7), the last *m*−*d* components of ${\hat{W}}^{⋄}$ are unaffected by $\Gamma_{d}$; the rotation only changes its first *m* components. Thus, under the adapted corr‐max transformation, the rotation of selected axes will leave some variables unchanged (those corresponding to unrotated axes) and the contributions of those variables to the quadratic form, as measured by the partition, will also be unchanged. We refer to this as the *rotation invariance property*.

Ideally, a partition yields orthogonal components that are closely related on a one‐to‐one basis with meaningful quantities. When these quantities cannot be the original *x* variables because of a collinearity, the rotation invariance property suggests that we might rotate the axes corresponding to variables involved in the collinearity, and then apply the transformation. There should still be close pairwise relationships between each unrotated variable and the variable to which it transforms, as these relationships are not compromised by the rotation. Also, there should now be close relationships between the quantities obtained through rotation and the variables to which they transform.

A rotation is attractive if it yields meaningful quantities. If, say, the only collinearity was between the first two variables, $X_{1}$ and $X_{2}$, a sensible rotation might be

$$\Gamma_{2}=\left[\begin{array}{cc} 2^{-1/2} & 2^{-1/2}\\ 2^{-1/2} & -2^{-1/2} \end{array}\right]\;\text{and}\;\Gamma =\left[\begin{array}{cc} \Gamma_{2} & 0\\ 0 & I_{m-2} \end{array}\right]\;\;,$$

which constructs two new variables, one proportional to the sum of $X_{1}$ and $X_{2}$, and the other proportional to their difference. This will often create variables that have a natural interpretation and the new variables will also have a low correlation if the variance of $X_{1}$ is similar to the variance of $X_{2}$. If rotation is used to counteract more than one distinct collinearity between the *x* variables, then $\Gamma_{m}$ should have a block diagonal form, with a separate block for each collinearity. An example is given in Section 5.2. When a collinearity involves more than two variables, constructing meaningful variables that have low correlations can be a challenging task. An approach based on orthogonal contrasts that might sometimes be useful is described in Appendix B.

While rotation can be helpful when collinearities are present, we should stress that rotation is never essential. The standard corr‐max transformation of Section 3 can be applied whenever **Σ** is a positive‐definite matrix, even if **Σ** contains high correlations, and it will yield a sensible partition of a quadratic form, as we discuss further in Section 7. Hence axes should only be rotated when the new variables that are constructed have an understandable interpretation.

## 5 Applications

In Section 5.1 we describe some common applications in which the corr‐max transformation yields a partition that quantifies the contributions of individual variables to a test statistic. In Section 5.2 an example is given in which collinearity is present and some *x*‐axes are rotated while applying the transformation.

### 5.1 Hotelling $T^{2}$, Mahalanobis distance and discriminant analysis

The standard application in which the partition is useful is where a statistic of interest, *Θ* say, has the form

(11) $$\Theta =\delta {\left(X-\mu \right)}^{⊤}{\hat{\Sigma }}^{-1}\left(X-\mu \right),$$

with $\hat{\Sigma }$ an estimate of **Σ**, var(***X***)∝**Σ** and *δ* a known positive scalar. From equation (5), the corr‐max transformation yields $\hat{W}={\left(\hat{D}\hat{\Sigma }\hat{D}\right)}^{-1/2}\hat{D}\left(X-\mu \right)$, and the contribution of the *i*th *x*‐variable to *Θ* is evaluated as $\delta w_{i}^{2}$, where ${\left(w_{1},\ldots ,w_{m}\right)}^{⊤}$ is the value of $\hat{W}$ given by data.

Before the partition can be applied, ***X***, $\hat{\Sigma }$, *δ*, and ***μ*** must be identified and it must be checked that var(***X***)∝**Σ**. (The matrix $\hat{D}$ is obtained from $\hat{\Sigma }$.) The individual contributions, $\delta w_{i}^{2}$ for *i*=1,…,*m*, then follow automatically. After using the transformation the analyst should examine the correlations between components of $\hat{W}$ and the corresponding components of **X**; rotation of *x*‐axes might be considered if some correlations are low. (In our examples we consider rotating axes when correlations are 0.8 or lower.)

In the following four applications, the first three have precisely the form given in (11), while the fourth is closely related to it.

- *Hotelling's one‐sample* $T^{2}$ *statistic*. A random sample of size *n* is taken from N(***μ***,**Σ**), giving a sample mean ***X*** and sample covariance ${\hat{\Sigma }}_{1}$. The standard test of the hypothesis $\mu =\mu_{0}$ is based on Hotelling's one‐sample $T^{2}$ statistic,
  (12) $$T_{1}^{2}=n{\left(\bar{X}-\mu_{0}\right)}^{⊤}{\hat{\Sigma }}_{1}^{-1}\left(\bar{X}-\mu_{0}\right).$$
  Let the role of ***X*** in (11) be played by ***X***, so that var(***X***)=**Σ**/*n*. The partition is obtained by putting $\hat{\Sigma }={\hat{\Sigma }}_{1}$, *δ*=*n* and $\mu =\mu_{0}$.
- *Hotelling's two‐sample* $T^{2}$ *statistic*. Two random samples of sizes $n_{1}$ and $n_{2}$ are drawn from the multivariate normal distributions, $N(\mu_{1},\Sigma )$ and $N(\mu_{2},\Sigma )$, that have the same covariance matrix. Then the hypothesis $\mu_{1}=\mu_{2}$ is tested using Hotelling's two‐sample $T^{2}$ statistic,
  (13) $$T_{2}^{2}=\left\{n_{1}n_{2}/\left(n_{1}+n_{2}\right)\right\}{\left({\bar{X}}_{1}-{\bar{X}}_{2}\right)}^{⊤}{\hat{\Sigma }}_{p}^{-1}\left({\bar{X}}_{1}-{\bar{X}}_{2}\right),$$
  where ${\bar{X}}_{1}$ and ${\bar{X}}_{2}$ are the sample means and ${\hat{\Sigma }}_{p}$ is the pooled estimate of **Σ** derived from the two samples. Let the role of ***X*** in (11) be played by ${\bar{X}}_{1}-{\bar{X}}_{2}$, so var(***X***)∝**Σ**. Put $\hat{\Sigma }={\hat{\Sigma }}_{p}$, $\delta =n_{1}n_{2}/\left(n_{1}+n_{2}\right)$ and ***μ***=**0** to obtain the contributions of individual variables to $T_{2}^{2}$.
- *Mahalanobis distance*. If $X_{\left(1\right)}$ and $X_{\left(2\right)}$ are two *m*×1 vectors, then the Mahalanobis distance between them is
  (14) $${\left(X_{\left(1\right)}-X_{\left(2\right)}\right)}^{⊤}{\hat{\Sigma }}^{-1}\left(X_{\left(1\right)}-X_{\left(2\right)}\right).$$
  Here $X_{\left(1\right)}$ and $X_{\left(2\right)}$ must be independent, but either or both of them could be individual observations, or sample means, or one of them could be a vector of known constants. We suppose $\text{var}\left(X_{\left(i\right)}\right)=k_{i}\Sigma$ (*i*=1,2) where $k_{1}$ or $k_{2}$ (but not both) may equal 0. We also suppose that $E(\hat{\Sigma })\propto \Sigma$ so, for example, $\hat{\Sigma }$ might be the maximum likelihood estimate or an unbiased estimate of **Σ**. Let $X=X_{\left(1\right)}-X_{\left(2\right)}$, so var(***X***)∝**Σ**. Put *δ*=1 and ***μ***=**0**. Then the partitioning gives the contributions of individual variables to the Mahalanobis distance.
- *Fisher's linear discriminant function*. Suppose an observation needs to be classified as belonging to one of two classes that are characterised by the multivariate normal distributions $N(\mu_{1},\Sigma )$ and $N(\mu_{2},\Sigma )$, with sample means ${\bar{X}}_{1}$ and ${\bar{X}}_{2}$ and common estimated covariance matrix ${\hat{\Sigma }}_{p}$. A new observation $X^{*}$ is classified as belonging to class 1 on the basis of Fisher's linear discriminant function if
  (15) $$r\left(X^{*}\right)={\left\{X^{*}-\frac{1}{2}\left({\bar{X}}_{1}+{\bar{X}}_{2}\right)\right\}}^{⊤}{\hat{\Sigma }}_{p}^{-1}\left({\bar{X}}_{1}-{\bar{X}}_{2}\right)>0.$$
  Consider the transformations
  (16) $${\hat{W}}^{\circ }={\left(\hat{D}{\hat{\Sigma }}_{p}\hat{D}\right)}^{-1/2}\hat{D}\left({\bar{X}}_{1}-{\bar{X}}_{2}\right)$$
  and
  (17) $${\hat{W}}^{*}={\left(\hat{D}{\hat{\Sigma }}_{p}\hat{D}\right)}^{-1/2}\hat{D}\left\{X^{*}-\frac{1}{2}\left({\bar{X}}_{1}+{\bar{X}}_{2}\right)\right\}.$$
  Since $\text{var}({\bar{X}}_{1}-{\bar{X}}_{2})\propto \Sigma$ and $\text{var}[X^{*}-\frac{1}{2}\left({\bar{X}}_{1}+{\bar{X}}_{2}\right)]\propto \Sigma$, Theorem 4 applies. Hence the *i*th components of both ${\hat{W}}^{\circ }$ and ${\hat{W}}^{*}$ can be identified with the *i*th *x*‐variable. Let $W_{i}^{\circ }$ and $W_{i}^{*}$ denote these components. Because $r\left(X^{*}\right)=\Sigma_{i=1}^{m}W_{i}^{\circ }W_{i}^{*}$, the contribution of the *i*th *x*‐variable to $r(X^{*})$ is given by the observed value of $W_{i}^{\circ }W_{i}^{*}$.

We use two examples to explore how the transformation and partition work in practice. In the first example we apply the transformation without rotation of variables and consider applications (a), (c) and (d). In the second example, given in the next subsection, we illustrate application (b) and apply the transformation to both rotated and un‐rotated variables.

Example 1 **Swiss bank notes** Flury & Riedwyl (1988) present data on 100 genuine Swiss 1000‐franc bank notes. Six measurements were made on each note: *length* (length), *left‐ht* (height measured on the left), *right‐ht* (height measured on the right), *lower* (distance from the inner frame to the lower border), *upper* (distance from the inner frame to the upper border), and *diagonal* (length of the diagonal). These measurements are the data values of $X={\left(X_{1},\ldots ,X_{6}\right)}^{⊤}$. Their sample standard deviations are (0.388, 0.364, 0.355, 0.643, 0.649, 0.447) and the reciprocals of these standard deviations form the diagonal entries of $\hat{D}$. The sample correlation matrix of **X** is:

(18) $$\hat{D}\hat{\Sigma }\hat{D}=\left(\begin{array}{cccccc} 1.000 & 0.411 & 0.416 & 0.229 & 0.057 & 0.032\\ 0.411 & 1.000 & 0.664 & 0.242 & 0.208 & -0.265\\ 0.416 & 0.664 & 1.000 & 0.255 & 0.133 & -0.150\\ 0.229 & 0.242 & 0.255 & 1.000 & -0.632 & -0.001\\ 0.057 & 0.208 & 0.133 & -0.632 & 1.000 & -0.260\\ 0.032 & -0.265 & -0.150 & -0.001 & -0.260 & 1.000 \end{array}\right)$$

where $\hat{\Sigma }$ is the sample covariance matrix. It can be seen that no correlation is larger than 0.664. The mean vector for the banknote measurements is $\bar{x}={\left(214.969,129.943,129.720,8.305,10.168,141.517\right)}^{⊤}$.

If the corr‐max transformation is applied to a vector ***X*** to yield a vector $\hat{W}$, the correlations between components of ***X*** and the corresponding components of $\hat{W}$ are equal to the diagonal entries of ${\left(\hat{D}\hat{\Sigma }\hat{D}\right)}^{1/2}$. These diagonal entries are 0.96, 0.90, 0.91, 0.91, 0.91 and 0.98. They are all large, indicating close one‐to‐one relationships between each *x*‐variable and its corresponding component of $\hat{W}$, so rotation of *x*‐variables is unnecessary.

Hotelling's one‐sample $T^{2}$ statistic might be used to test the hypothesis that the population mean vector is, say, $\mu_{0}=\;{\left(215.007,129.979,129.756,8.369,10.233,\;141.562\right)}^{⊤}$. These values have been chosen so that, for each variable, the hypothesised population mean exceeds the sample mean by 0.1 standard deviations. The value of the test statistic, given by equation (12), is $T_{1}^{2}=8.74$. We have already calculated $\hat{D}$ and $\hat{D}\hat{\Sigma }\hat{D}$. Setting ***X*** and ***μ*** equal to **x** and $\mu_{0}$ respectively in equation (5) gives $\hat{W}=-{\left(0.051,0.053,0.055,0.164,0.182,0.138\right)}^{⊤}$. As *δ*=100, the contribution of the *i*th *x*‐variable to $T_{1}^{2}$ is $100w_{i}^{2}$, so the contributions of the six *x*‐variables are $0.51^{2}$, $0.53^{2}$, $0.55^{2}$, $1.64^{2}$, $1.82^{2}$ and $1.38^{2}$. These values sum to 8.75, which differs slightly from $T_{1}^{2}$ because we have listed all contributions to 2 decimals only, and not given their precise values. The actual sum of the contributions equals $T_{1}^{2}$ as the theory tells us. Although for each component the sample mean differs from the hypothesised population mean by an equivalent amount, the last three *x*‐variables (*lower, upper,* and *diagonal*) make larger contributions to the $T_{1}^{2}$ statistic than the first three *x*‐variables (*length*,* left‐ht* and *right‐ht*).

As an example involving Mahalanobis distance, suppose the measurements for an additional banknote that might be a forgery are $x_{2}={\left(215.8,129.7,129.0,6.9,8.6,143.2\right)}^{⊤}$. The Mahalonobis distance between $x_{2}$ and the mean value of ***X*** in the sample of 100 genuine banknotes **x**, is given by equation (14) with $X_{\left(1\right)}=\bar{x}$ and $X_{\left(2\right)}=x_{2}$. The value of this distance is 55.69, which gives clear evidence the note is a forgery (*p*<0.0001). Our partition can be used to determine which characteristics of the new banknote distinguish it from the genuine banknotes. We put $X=\bar{x}-x_{2}$ and ***μ***=**0** in equation (5), to obtain $\hat{W}$. As *δ*=1, the contribution of the *i*th *x*‐variable to the Mahalanobis distance is the square of the *i*th component of $\hat{W}$. These squared values are (8.64 0.87 4.54 16.66 15.12 9.86). Hence the measurements that most distinguish the new banknote from genuine banknotes are $X_{4}$ (*lower*) and $X_{5}$ (*upper*).

The Swiss bank notes dataset given by Flury & Riedwyl (1988) contained 100 faked bank notes in addition to the 100 genuine notes. As an example that involves Fisher's discriminant rule, we consider the task of using these data to classify a note as *genuine* or from the same popultation as the *fakes*. The pooled sample covariance matrix based on all 200 notes is

(19) $${\hat{\Sigma }}_{p}=\left(\begin{array}{cccccc} 0.1371 & 0.0448 & 0.0406 & -0.0217 & 0.0169 & 0.0085\\ 0.0448 & 0.0988 & 0.0663 & 0.0163 & 0.0186 & -0.0241\\ 0.0406 & 0.0663 & 0.1076 & 0.0198 & 0.0154 & 0.0052\\ -0.0217 & 0.0163 & 0.0198 & 0.8473 & -0.3768 & 0.1191\\ 0.0169 & 0.0186 & 0.0154 & -0.3768 & 0.4128 & -0.0487\\ 0.0085 & -0.0241 & 0.0052 & 0.1191 & -0.0487 & 0.2555 \end{array}\right)\;\;.$$

Table 1 summarises the analysis. The first two rows, ${\bar{X}}_{1}$ and ${\bar{X}}_{2}$, show the sample means of the genuine and faked bank notes, respectively. The note to be classified is $X^{*}$. Equation (15) gives −20.34 as the value of $r(X^{*})$, indicating that the new note should be classified as coming from the same population as the fakes. Applying equations (16) and (17) we obtain ${\hat{W}}^{\circ }$ and ${\hat{W}}^{*}$ (fourth and fifth rows). The contributions of individual *x*‐variables to $r(X^{*})$ are evaluated as the diagonal entries of ${\hat{W}}^{\circ }{\left({\hat{W}}^{*}\right)}^{⊤}$, shown in the last row. Unlike the previous two examples, some of these values are negative; negative values suggest the new note is from the same population as the faked notes. The last three variables, *lower*,* upper,* and *diagonal*, underlie the outcome of the discrimination rule, as they make much larger contributions to $r(X^{*})$ (in absolute value) than the first three variables.

**Table 1:** Values from the discriminant analysis for a Swiss bank note

	$X_{1}$	$X_{2}$	$X_{3}$	$X_{4}$	$X_{5}$	$X_{6}$
${\bar{X}}_{1}$	214.969	129.943	129.720	8.305	10.168	141.517
${\bar{X}}_{2}$	214.823	130.300	130.193	10.530	11.133	139.45
$X^{*}$	214.4	130.1	130.3	9.7	11.7	139.8
${\hat{W}}^{\circ }$	0.38	−0.001	−1.48	−4.16	−2.80	4.56
${\hat{W}}^{*}$	−1.44	−0.64	1.49	1.21	2.10	−1.46
${\left[{\hat{W}}^{\circ }{\left({\hat{W}}^{*}\right)}^{⊤}\right]}_{ii}$	−0.55	0.001	−2.21	−5.04	−5.89	−6.67

In Section 1 we noted that Rotenberry *et al*. (2006) examined eigenvectors corresponding to small eigenvalues in order to determine influential variables on a Mahalanobis distance. Before leaving this example we illustrate their method by applying it to the case where we have 100 genuine banknotes and an additional banknote that might be a forgery. Their approach is to decompose the quadratic form $Q={\left(\bar{x}-x_{2}\right)}^{⊤}{\hat{\Sigma }}^{-1}\left(\bar{x}-x_{2}\right)$ as

$$Q=\sum_{j=1}^{m}u_{j}^{2}/\lambda_{j}$$

where $u_{j}={\left(\bar{x}-x_{2}\right)}^{⊤}\gamma_{j}$, $\lambda_{1}>\ldots >\lambda_{m}$ are the eigenvalues of $\hat{\Sigma }$ and $\gamma_{1},\ldots ,\gamma_{m}$ are the corresponding eigenvectors. They focus on small eigenvalues because, if $\lambda_{j}$ is small, then $X^{⊤}\gamma_{j}$ varies little for the *X* values in the hundred genuine banknotes, so that a large value of ${\left(\bar{x}-x_{2}\right)}^{⊤}\gamma_{j}$ is more indicative of forgery. The eigenvalues of the sample covariance matrix of the genuine banknotes are 0.69, 0.36, 0.19, 0.087, 0.080 and 0.041, so interest centres on either just the smallest eigenvalue or the smallest three eigenvalues. The following are the eigenvectors for the three smallest eigenvalues:

**Table:** 

	*length*	*left‐ht*	*right‐ht*	*lower*	*upper*	*diagonal*
Smallest	−0.011	0.737	−0.666	−0.050	−0.062	0.072
2${}^{nd}$ smallest	0.113	−0.360	−0.481	0.559	0.548	0.116
3${}^{rd}$ smallest	0.786	−0.243	−0.280	−0.243	−0.244	−0.354

Based on just the eigenvector corresponding to the smallest eigenvalue, $X_{2}$ and $X_{3}$ (*left‐ht* and *right‐ht*) are clearly the most important variables, since in the case of that eigenvector they have much larger coefficients (in absolute value) than the other variables. However, if the three eigenvectors displayed above are all considered relevant, then deciding which *x*‐variables are important is not clear‐cut and requires the analyst to make an intuitive judgment. Moreover, there is no obvious method of determining the relative quantitative importance of different variables, and with any such method the answers are likely to depend on whether the three smallest eigenvalues or only the very smallest are considered “small”.

### 5.2 Collinearities, rotation and quadratic forms

Some advantages of the (un‐adapted) corr‐max transformation are diminished when strong collinearities are present: not every *X* variable will be closely related to the transformed variable with which it is paired. Here we examine a dataset in which collinearities are present and illustrate use of the cos‐max transformation matrix to identify the variables that are collinear. To identify collinearities we apply the cos‐max transformation to data that have been standardised to have means of 0 and variances of 1, making the cos‐max and corr‐max transformations very similar, as will be seen.

The dataset contains two strata whose means will be compared using Hotelling's two‐sample $T^{2}$ statistic. We partition the test statistic into the contributions of individual variables/variable combinations by applying the adapted corr‐max transformation. The rotation matrix (**Γ**) we use in the transformation creates meaningful non‐collinear variables from the variables that are involved in the collinearities.

Example 2 **Female and male athletes** The data relate to the following nine measurements $\left(X_{1},\ldots ,X_{9}\right)$ that were made on $n_{1}=100$ female and $n_{2}=102$ male athletes collected at the Australian Institute of Sport (Cook & Weisberg 1994): *Wt* (weight), *Ht* (height), *Rcc* (red blood cell count), *Hg* (hemoglobin), *Hc* (hematocrit), *Wcc* (white blood cell count), *Ferr* (plasma ferratin concentration), *Bfat* (% body fat), and *SSF* (sum of skin folds). It is assumed that the two groups (females and males) may have different means, $\mu_{1}$ and $\mu_{2}$, but have a common covariance matrix **Σ**. Let ${\hat{\Sigma }}_{p}$ denote the pooled estimate of **Σ**. The pooled correlation matrix, $\hat{D}{\hat{\Sigma }}_{p}\hat{D}$, takes the value

(20) $$\left(\begin{array}{ccccccccc} 1.00 & 0.68 & 0.05 & 0.10 & 0.06 & 0.15 & 0.06 & 0.63 & 0.65\\ 0.68 & 1.00 & -0.04 & -0.11 & -0.04 & 0.05 & -0.15 & 0.34 & 0.34\\ 0.05 & -0.04 & 1.00 & 0.78 & 0.86 & 0.14 & -0.05 & -0.04 & -0.05\\ 0.10 & -0.11 & 0.78 & 1.00 & 0.90 & 0.13 & 0.01 & -0.04 & -0.06\\ 0.06 & -0.04 & 0.86 & 0.90 & 1.00 & 0.15 & -0.06 & -0.08 & -0.11\\ 0.15 & 0.05 & 0.14 & 0.13 & 0.15 & 1.00 & 0.12 & 0.21 & 0.21\\ 0.06 & -0.15 & -0.05 & 0.01 & -0.06 & 0.12 & 1.00 & 0.16 & 0.16\\ 0.63 & 0.34 & -0.04 & -0.04 & -0.08 & 0.21 & 0.16 & 1.00 & 0.97\\ 0.65 & 0.34 & -0.05 & -0.06 & -0.11 & 0.21 & 0.16 & 0.97 & 1.00 \end{array}\right)\;\;$$

Under the cos‐max transformation, a data matrix **X** is transformed to ${\left(X^{⊤}X\right)}^{-1/2}X$. Let $X_{s}$ denote the data matrix after variables have been centred and scaled so that the correlation matrix of $X_{s}$ is $X_{s}^{⊤}X_{s}$. If we put ${\left(X_{s}^{⊤}X_{s}\right)}^{-1/2}=H={\left(h_{1},\ldots ,h_{m}\right)}^{⊤}$ then, as Garthwaite *et al*. (2012) pointed out, the variance inflation factor for the *j*th variable (${VIF}_{j}$) is equal to $h_{j}^{⊤}h_{j}$. Moreover, if ${VIF}_{j}$ is large, indicating a collinearity, then large components of $h_{j}$ correspond to the variables that underlie the collinearity. In the present example, $X_{s}^{⊤}X_{s}=\hat{D}{\hat{\Sigma }}_{p}\hat{D}$, so examining the rows of ${\left(\hat{D}{\hat{\Sigma }}_{p}\hat{D}\right)}^{-1/2}$ identifies variables involved in collinearities. (When $X_{s}^{⊤}X_{s}=\hat{D}{\hat{\Sigma }}_{p}\hat{D}$, the corr‐max and cos‐max transformations are the same.)

We put ${\left(\hat{D}{\hat{\Sigma }}_{p}\hat{D}\right)}^{-1/2}={\left(h_{1},\ldots ,h_{m}\right)}^{⊤}$ and give the values of the $h_{j}^{⊤}$ in Table 2. Values greater than 0.80 in absolute value are given in bold‐face type. The last column of the table gives the VIF for each variable, e.g. 8.15 is the VIF for $X_{5}$ and equals $h_{5}^{⊤}h_{5}$. A VIF above 10 is often treated as indicative of collinearity (Neter, Wasserman & Kutner 1983 p. 392) On this basis, $X_{8}$ (*Bfat*) and $X_{9}$ (*SSF*) are involved in collinearites and, from the bold‐face numbers in the display of $h_{8}$ and $h_{9}$, there is a collinearity between them. Weaker boundaries for flagging a collinearity have also been proposed; Menard (1995, p. 66) suggests a VIF above 5 should raise concern and O'Brien (2007) reports that boundary values as low as 4 have been suggested as rules of thumb. A boundary of 4 or 5 would indicate one further collinearity, between $X_{4}$ (*Hg*) and $X_{5}$ (*Hc*).

**Table 2:** Rows of ${\left(\hat{D}{\hat{\Sigma }}_{p}\hat{D}\right)}^{-1/2}$ and variance inflation factors for data on athletes

	$X_{1}$	$X_{2}$	$X_{3}$	$X_{4}$	$X_{5}$	$X_{6}$	$X_{7}$	$X_{8}$	$X_{9}$	VIF
$h_{1}^{⊤}$	**1.66**	−0.61	0.06	−0.23	0.00	−0.01	−0.06	−0.17	−0.41	3.38
$h_{2}^{⊤}$	−0.61	**1.36**	−0.03	0.23	−0.08	0.00	0.14	−0.06	0.07	2.31
$h_{3}^{⊤}$	0.06	−0.03	**1.75**	−0.30	−0.80	−0.03	0.02	0.05	−0.10	3.83
$h_{4}^{⊤}$	−0.23	0.23	−0.30	**2.09**	−**1.12**	0.00	−0.04	0.04	0.00	5.82
$h_{5}^{⊤}$	0.00	−0.08	−0.80	−**1.12**	**2.48**	−0.08	0.06	−0.09	0.22	8.15
$h_{6}^{⊤}$	−0.01	0.00	−0.03	0.00	−0.08	**1.04**	−0.05	−0.07	−0.06	1.09
$h_{7}^{⊤}$	−0.06	0.14	0.02	−0.04	0.06	−0.05	**1.04**	−0.07	−0.02	1.11
$h_{8}^{⊤}$	−0.17	−0.06	0.05	0.04	−0.09	−0.07	−0.07	**3.27**	−**2.43**	16.64
$h_{9}^{⊤}$	−0.41	0.07	−0.1	0.00	0.22	−0.06	−0.02	−**2.43**	**3.38**	17.59

If the corr‐max transformation is applied to $X={\left(X_{1},\ldots ,X_{9}\right)}^{⊤}$, then the following are the sample correlations between each *x* variable and the variable to which it transforms:

**Table:** 

Variable:	*Wt*	*Ht*	*Rcc*	*Hg*	*Hc*	*Wcc*	*Ferr*	*Bfat*	*SSF*
Correlation:	0.84	0.91	0.83	0.80	0.76	0.99	0.99	0.76	0.75

The correlations for *Hg* and *Hc* are a little low, suggesting that remedial action might be taken to offset both the mild collinearity between this pair of variables as well as the stronger collinearity between *Bfat* and *SSF*. To rotate the axes associated with these variable pairs we replace the corr‐max transformation by the adapted corr‐max transformation given by equation (9), with **Γ** set equal to the following block‐diagonal orthogonal matrix:

(21) $$\Gamma =\left(\begin{array}{ccccccccc} 1 & 0 & 0 & 0 & 0 & 0 & 0 & 0 & 0\\ 0 & 1 & 0 & 0 & 0 & 0 & 0 & 0 & 0\\ 0 & 0 & 1 & 0 & 0 & 0 & 0 & 0 & 0\\ 0 & 0 & 0 & 2^{-1/2} & 2^{-1/2} & 0 & 0 & 0 & 0\\ 0 & 0 & 0 & 2^{-1/2} & -2^{-1/2} & 0 & 0 & 0 & 0\\ 0 & 0 & 0 & 0 & 0 & 1 & 0 & 0 & 0\\ 0 & 0 & 0 & 0 & 0 & 0 & 1 & 0 & 0\\ 0 & 0 & 0 & 0 & 0 & 0 & 0 & 2^{-1/2} & 2^{-1/2}\\ 0 & 0 & 0 & 0 & 0 & 0 & 0 & 2^{-1/2} & -2^{-1/2} \end{array}\right)\;\;.$$

We refer to the variables to which *Bfat* and *SSF* transform as *B*+*S* and *B*−*S*, and those from *Hg* and *Hc* as *H*+*H* and *H*−*H*. Rotation dramatically increased the correlations between the new variables and their transformed values while leaving the corresponding correlations of all other variables unchanged. The correlations between the rotated *x* variables and the variables to which they transform are displayed below. These show a close one‐to‐one relationship between the two sets of variables.

**Table:** 

Variable:	Wt	Ht	Rcc	H+H	H‐H	Wcc	Ferr	B+S	B‐S
Correlation:	0.84	0.91	0.83	0.91	0.96	0.99	0.99	0.95	0.99

Before rotation, the sample means for the female and male athletes (${\bar{x}}_{1}$ and ${\bar{x}}_{2}$), and the pooled standard deviations (S.D.) were as follows.

**Table:** 

	Wt	Ht	Rcc	Hg	Hc	Wcc	Ferr	Bfat	SSF
Female:	67.34	174.59	4.405	13.560	40.48	6.994	57.0	17.85	87.0
Male:	82.52	185.51	5.027	15.553	45.65	7.221	96.4	9.25	51.4
S. D.	11.69	8.07	0.336	0.929	2.60	1.801	43.3	4.45	27.3

The reciprocals of the standard deviations constitute the non‐zero (diagonal) entries of the matrix $\hat{D}$. When Hotelling's $T^{2}$ test is used to compare the means of the two groups we obtain a $T_{2}^{2}$ statistic equal to 1199.1. This value gives, as you might expect, very clear evidence of differences between the two groups (p≃0.0000). However, the question of which quantities contribute most to the $T_{2}^{2}$ value is still relevant.

Putting ${\hat{w}}^{⋄}=\Gamma {\left(\hat{D}{\hat{\Sigma }}_{p}\hat{D}\right)}^{-1/2}\hat{D}\left({\bar{x}}_{1}-{\bar{x}}_{2}\right)$ gives

$${\hat{w}}^{⋄}=\left(-1.75,-1.48,-1.08,-1.74,-0.49,-0.06,-1.28,2.41,2.57\right).$$

The partition allows us to evaluate the contributions of individual *x*‐variables/variable combinations to $T_{2}^{2}$ as proportional to the squares of the components of ${\hat{w}}^{⋄}$:

3.08,2.19,1.17,3.02,0.24,0.00,1.64,5.81,6.61.

(When multiplied by *δ*, which here equals 100(102)/(100+102), these sum to the value of the $T_{2}^{2}$ statistic, 1199.1, apart from rounding error.) On the scale given by our partition, the largest contributors to the size of $T^{2}$ are the average of *Bfat* and *SSF* (contributing 24%) and the difference between these same two quantities (contributing 28%). With the other pair of variables whose axes were rotated, *Hg* and *Hc*, their average makes a substantial contribution (13%) while the contribution from their difference is only 1%.

## 6 Bootstrap confidence intervals

The corr‐max transformation gives point estimates of the contributions of individual variables to a quadratic form. Obtaining theoretical results that give interval estimates of these contributions is difficult, but the bootstrap can be used to obtain approximate confidence intervals. We elucidate the procedure through examples.

### 6.1 Confidence interval for contributions to a Mahalanobis distance

In Example 1 there were 100 genuine Swiss 1000‐franc bank notes and an additional bank note that might be a forgery. The Mahalanobis distance between the potential forgery and the mean of the genuine bank notes was 55.69 and the contributions of the six individual variables were estimated as (8.64 0.87 4.54 16.66 15.12 9.86). To obtain bootstrap confidence intervals for these contributions we generated 100 000 resamples from the 100 genuine bank notes. Each resample was a random sample of size 100 drawn *with replacement* from the 100 genuine notes.

Each resample was used in the same way as the original sample. The Mahalanobis distance between the potential forgery and the mean of the resample was calculated, with the resample being used to estimate the covariance matrix, $\hat{\Sigma }$. The contributions of individual variables to the Mahalanobis distance were then evaluated using the corr‐max transformation. This gave 100 000 estimates of the contribution of each variable and the *k*th smallest of these is equated to the (*k*/1000)th percentile of the bootstrap distribution. The median for a variable's contribution is thus the 50 000th smallest value and the endpoints of an approximate 95% confidence interval are the 2500th smallest and 2500th largest values. (This is the simplest method of forming bootstrap confidence intervals. As is well known, it typically works reasonably well but produces some bias, so work is underway to explore its performance in the current context and compare it with other bootstrap methods.)

**Figure 1:**
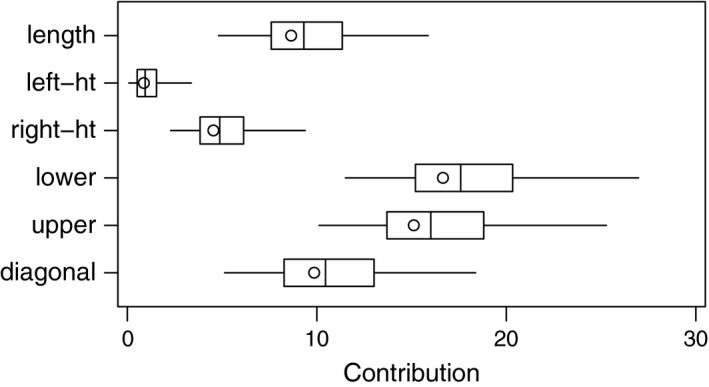
Confidence intervals for the contributions of individual variables to the Mahalanobis distance of a potential banknote forgery.

Figure 1 gives ‘pseudo‐boxplots’ for the contributions of each of the six variables. As in a conventional boxplot, the ends of the box indicate the interquartile range of the data and the line within the box marks the median. However, we used the whiskers to depict the central 95% confidence interval, rather than the trimmed range. The circles show the point estimates (8.64,…,9.86) given by the actual data. The figure indicates that measurements of the height on the left and right sides (left‐ht and right‐ht) contribute comparatively little to the Mahalanobis distance, while the distances from the inner frame to the lower border (lower) and from the inner frame to the upper border (upper) contribute noticeably more. Other firm conclusions are difficult to make, because there is substantial uncertainty as to the contributions of variables.

### 6.2 Confidence interval for contributions to a two‐sample $T^{2}$ statistic

Example 2 involves the study of a group of 100 female athletes and a group of 102 male athletes. Nine variables were measured on each athlete and two pairs of variables were rotated to reduce collinearities. The $T_{2}^{2}$ statistic for comparing the two groups was calculated and gave overwhelming evidence that the groups differed. To form bootstrap confidence intervals for the contributions of individual variables to this statistic, the groups must be resampled separately ‐ a resample consists of the measurements of 100 athletes randomly drawn with replacement from the female athletes and 102 athletes drawn with replacement from the male athletes. The $T_{2}^{2}$ statistic was determined for each of 100 000 resamples and the contribution of individual variables/variable combinations to the statistic in each resample was evaluated using the adapted corr‐max transformation.

**Figure 2:**
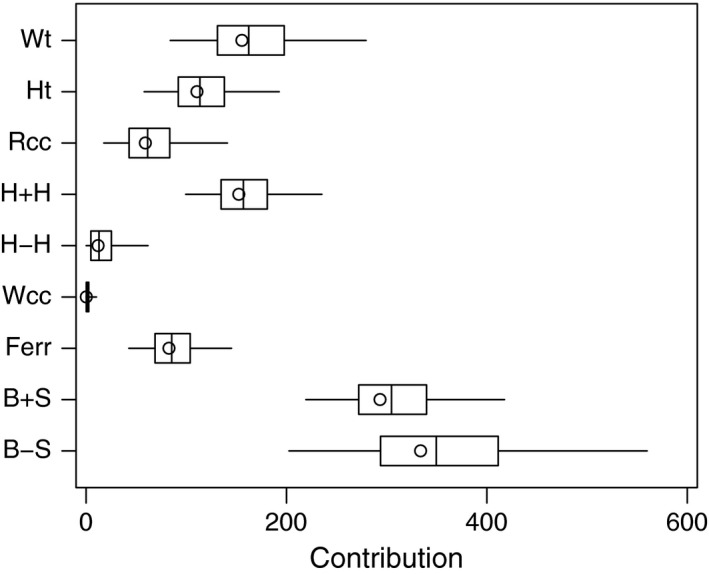
Confidence intervals for the contributions of individual variables to a two‐sample $T^{2}$ statistic for comparing female and male athletes.

Pseudo boxplots derived from the results are given in Figure 2. These show that the primary contributions to the $T^{2}$ statistic are clearly from *B*+*S* and *B*−*S*, the combination variables that are formed from the sums and differences of *Bfat* (percentage of body fat) and *SSF* (sum of skin folds). Other variables contribute noticeably less, but the only variable that clearly makes almost no contribution is the white cell blood count (*Wcc*). The confidence intervals are skewed to the right and the larger contributions tend to have wider confidence intervals. These appear to be characteristic traits and can also be seen in Figure 1.

## 7 Concluding comments

The corr‐max transformation is straightforward to calculate. The matrices $\hat{\Sigma }$ and $\hat{D}$ are readily determined and a spectral decomposition gives, say, $\hat{D}\hat{\Sigma }\hat{D}=H\Psi H^{⊤}$ where **Ψ** is a diagonal matrix of eigenvalues of $\hat{D}\hat{\Sigma }\hat{D}$ and **H** is an orthogonal matrix whose columns are eigenvectors. After **Ψ** and $\hat{\Sigma }$ have been determined, ${\left(\hat{D}\hat{\Sigma }\hat{D}\right)}^{-1/2}$ is set equal to $H\Psi^{-1/2}H^{⊤}$. Hence, the corr‐max transformation and the partition it yields are readily implemented in any programming language that offers matrix functions. To facilitate use of the partition in some important applications, programs have been written in R that determine the contributions of individual variables to a quadratic form in the contexts of Hotelling's one‐sample and two‐sample *T* ${}^{2}$ tests, Mahalanobis distance, and the classification of an item to one of two populations on the basis of Fisher's linear discriminant function. These programs are available from URL: http://users.mct.open.ac.uk/paul.garthwaite.

The rotation of variables has received much attention in this paper, and further comment is needed to give a balanced perspective on its role in partitioning a quadratic form. As in equation (5), let $\hat{W}={\left(\hat{D}\hat{\Sigma }\hat{D}\right)}^{-1/2}\hat{D}\left(X-\mu \right)$. When the correlations are high between each component of $\hat{W}$ and the corresponding component of ***X***, then the partition is clearly a sensible way of evaluating the contribution of each *x*‐variable. When some of these correlations are low, they can sometimes be increased dramatically through rotations that yield interpretable variables. This potential benefit of rotation was illustrated in Section 5.2. However, finding suitable rotations that yield interpretable variables is not always possible. Moreover, even when such rotations can be found, there are attractions in the simplicity of forming a partition that retains the original *x*‐variables. We briefly return to the athletes data to show that low correlations do not preclude a transparent relationship between the *x*‐variables and the contributions allocated to them by the partition.

Let $X^{\#}$ denote the difference between an athlete's measurements and the average for their gender. Put $X^{*}=\hat{D}X^{\#}$, so that the components of $X^{*}=\left(X_{1}^{*},\ldots ,X_{9}^{*}\right)$ are standardized values of each variable. Let ${\left({\hat{W}}_{1},\ldots ,{\hat{W}}_{9}\right)}^{⊤}=\hat{W}={\left(\hat{D}{\hat{\Sigma }}_{p}\hat{D}\right)}^{-1/2}X^{*}$. Then $\delta {\hat{W}}_{i}^{2}$ is the contribution of the *i*th variable to the quadratic form **Θ** in equation (11). We focus on the two most highly correlated variables, *Bfat* $\left(X_{8}\right)$ and *SSF* $\left(X_{9}\right)$. The partition uses the following equations (obtained from Table 2) to determine their contribution to **Θ**.

$$\begin{array}{cc} & {\hat{W}}_{8}^{2}=\left(\right.-0.17X_{1}^{*}-0.06X_{2}^{*}+0.05X_{3}^{*}+0.04X_{4}^{*}-0.09X_{5}^{*}-0.07X_{6}^{*}\\ & \;-0.07X_{7}^{*}+3.27X_{8}^{*}-2.43X_{9}^{*}{)}^{2}\\ & {\hat{W}}_{9}^{2}=\left(\right.-0.41X_{1}^{*}+0.07X_{2}^{*}-0.10X_{3}^{*}+0.00X_{4}^{*}+0.22X_{5}^{*}-0.06X_{6}^{*}\\ & \;-0.02X_{7}^{*}-2.43X_{8}^{*}+3.38X_{9}^{*}{)}^{2} \end{array}$$

These formulae show precisely how the contributions of individual variables to *Θ* are calculated. In particular, the formulae show that the difference between $X_{8}^{*}$ and $X_{9}^{*}$ has a substantial impact on the assessed contributions of *Bfat* and *SSF*. This arises from the high correlation between them (the correlation is 0.96), so that a large difference between their standardised differences is unexpected and so increases *Θ*. The role of the interaction between *Bfat* and *SSF* can be further clarified by writing ${\hat{W}}_{8}^{2}$ and ${\hat{W}}_{9}^{2}$ as,

(22) $${\hat{W}}_{8}^{2}={\left\{-0.17X_{1}^{*}-0.06X_{2}^{*}+0.05X_{3}^{*}+0.04X_{4}^{*}-0.09X_{5}^{*}-0.07X_{6}^{*}-0.07X_{7}^{*}+0.84X_{8}^{*}+2.43\left(X_{8}^{*}-X_{9}^{*}\right)\right\}}^{2}$$

(23) $${\hat{W}}_{9}^{2}={\left\{-0.41X_{1}^{*}+0.07X_{2}^{*}-0.10X_{3}^{*}+0.00X_{4}^{*}+0.22X_{5}^{*}-0.06X_{6}^{*}-0.02X_{7}^{*}+2.43\left(X_{9}^{*}-X_{8}^{*}\right)+0.95X_{9}^{*}\right\}}^{2}$$

Written in this way, ${\hat{W}}_{8}^{2}$ and ${\hat{W}}_{9}^{2}$ seem a very reasonable reflection of the respective contributions of *Bfat* and *SSF* to the quadratic form, inasmuch as the large terms in (22) both involve $X_{8}^{*}$ while those in (23) both involve $X_{9}^{*}$. We should note though, that while our method gives contributions to ${\hat{W}}_{8}^{2}$ and ${\hat{W}}_{9}^{2}$ that seem reasonable, other methods may give different values that also seem reasonable. We should also note that, in this example, the low correlation on which we focused stems from a single collinearity between just two variables. With multiple collinearities involving several variables, the relationship between the *x*‐variables and the contributions allocated to them would be less straightforward.

Experiments are often laborious and costly to conduct and the scientists who conduct them would like to glean as much as possible from the data they gather. Not infrequently, a quadratic form is central to a multivariate statistical analysis and then the scientists might reasonably expect the quadratic form to yield more than just a *p*‐value from a hypothesis test. The method developed in this paper provides a means of learning more about a quadratic form and hence should prove useful. It can always be applied, provided that $\hat{\Sigma }$ is positive‐definite, and yields a well‐defined numerical evaluation of the contributions of individual *x*‐variables to the quadratic farm. With multiple collinearities involving several variables, it can be difficult to judge intuitively whether an evaluation is a sensible reflection of these contributions and then the credibility of an evaluation must stem from the method used to produce it. Our method is derived from a clear, understandable criterion that gives it a sound basis. In our experience the method has never given an evaluation that seems unreasonable and we recommend its use for the decomposition of a quadratic form for any positive‐definite matrix $\hat{\Sigma }$. In reporting results, the method used to obtain the decomposition should be stated so as to define the evaluated contributions unambiguously.

## Appendix A Proofs of theorems

Lemma 1 Suppose that **B** is a square matrix and *tr*(**B**) is to be maximised under the condition that $B^{⊤}B=\Omega$, where **Ω** is a positive‐definite matrix. Then $B=\Omega^{1/2}$, the symmetric square‐root of **Ω**.

Because **Ω** is positive‐definite, **B** is of full rank, whence the singular value decomposition theorem gives $B=F\Lambda^{1/2}G^{⊤}$, where **Λ** is a diagonal matrix and **F** and **G** are orthogonal matrices. Then $B^{⊤}B=G\Lambda G^{⊤}$, so $G\Lambda G^{⊤}$ is the unique spectral decomposition of **Ω**. Also, $max\left\{tr(B)\right\}=max\left\{tr(F\Lambda^{1/2}G^{⊤})\right\}=max\left\{tr(\Lambda^{1/2}G^{⊤}F)\right\}$. Now **G** and **F** are orthogonal matrices, so the maximum value that the (*i*,*i*)th entry of $G^{⊤}F$ can take is 1, and it can only equal 1 if the *i*th columns of **G** and **F** are equal. Hence $tr(\Lambda^{1/2}G^{⊤}F)$ is maximised when **G**=**F**. Thus $B=G\Lambda^{1/2}G^{⊤}=\Omega^{1/2}$.

Lemma 2 Suppose that E(***Y***)=**0** and $\text{var}\left(Y\right)\propto \Omega^{-1}$, and that $E(Y^{⊤}BY)$ is to be maximised, where **B** is a square matrix and $B^{⊤}B=\Omega$. Then $B=\Omega^{1/2}$.

Let $\text{var}\left(Y\right)=k\Omega^{-1}$ where *k* is a scalar. Observe that $E\left(Y^{⊤}BY\right)=E\left[tr\left(Y^{⊤}BY\right)\right]=E\left[tr\left(BYY^{⊤}\right)\right]=tr\left[BE\left(YY^{⊤}\right)\right]=ktr\left[B\Omega^{-1}\right]=ktr\left[B{\left(B^{⊤}B\right)}^{-1}\right]=ktr\left[{\left(B^{⊤}\right)}^{-1}\right]$. Hence we wish to maximise $tr[{\left(B^{⊤}\right)}^{-1}]$ under the constraint that $B^{⊤}B=\Omega$ or, equivalently, that $B^{-1}{\left(B^{⊤}\right)}^{-1}=\Omega^{-1}$. From Lemma 1, ${\left(B^{⊤}\right)}^{-1}=\Omega^{-1/2}$, so $B=\Omega^{1/2}$.

Proof of Theorem 1 For any ***X***, by assumption ${\left(X-\mu \right)}^{⊤}\Sigma^{-1}\left(X-\mu \right)=W^{⊤}W={\left(X-\mu \right)}^{⊤}A^{⊤}A\left(X-\mu \right)$. Choosing ***X*** so that only one entry of (***X***−***μ***) is non‐zero shows that the diagonal entries of $\Sigma^{-1}$ and $A^{⊤}A$ are equal. Choosing ***X*** so that only two entries of (***X***−***μ***) are non‐zero then does the same for off‐diagonal entries, so $\Sigma^{-1}=A^{⊤}A$. Since var(***X***)∝**Σ**, it follows that $\text{var}\left(W\right)\propto A\Sigma A^{⊤}=A{\left(A^{⊤}A\right)}^{-1}A^{⊤}=I$. Consequently the components of **W** are uncorrelated and have identical variances. For the next part of the theorem, let $W^{*}=A\left\{X-E(X)\right\}$ and var(***X***)=*k* **Σ**. Then $E(W^{*})=0$ and $\text{var}\left(W^{*}\right)=kA\Sigma A^{⊤}=kA{\left(A^{⊤}A\right)}^{-1}A^{⊤}=kI$. Let **Z**=**D**{***X***−E(**X**)} and put $Z={\left(Z_{1},\ldots ,Z_{m}\right)}^{⊤}$, so that E(**Z**)=0, var(**Z**)=*k* **DΣD**, and $\text{var}(Z_{i})=k$ for *i*=1,…,*m*. We know that $\text{cor}\left(X_{i},W_{i}^{*}\right)=\text{cor}\left(Z_{i},W_{i}^{*}\right)=E\left(Z_{i}W_{i}^{*}/k\right)$. In addition cor$\left(X_{i},W_{i}\right)=\text{cor}\left(X_{i},W_{i}^{*}\right)$ because $W-W^{*}=A\left\{E(X)-\mu \right\}$. Thus $\Sigma_{i=1}^{m}\text{cor}\left(X_{i},W_{i}\right)=E\left(Z^{⊤}W^{*}/k\right)=E\left(Z^{⊤}AD^{-1}Z/k\right)$. Also the constraint $A^{⊤}A=\Sigma^{-1}$ is equivalent to ${\left(AD^{-1}\right)}^{⊤}\left(AD^{-1}\right)=D^{-1}\Sigma^{-1}D^{-1}$. Hence **A** must be chosen to maximise $E(Z^{⊤}AD^{-1}Z)$, where E(**Z**)=0, var(**Z**)∝**DΣD** and ${\left(AD^{-1}\right)}^{⊤}\left(AD^{-1}\right)={\left(D\Sigma D\right)}^{-1}$. From Lemma 2, $AD^{-1}={\left(D\Sigma D\right)}^{-1/2}$. Thus, $A={\left(D\Sigma D\right)}^{-1/2}D$ and $W={\left(D\Sigma D\right)}^{-1/2}\times D\left(X-\mu \right)$.

Proof of Theorem 3 Let var(*X*)=*k* **Σ**,** Z**=**D**{**X**−E(**X**)} and put $Z={\left(Z_{1},\ldots ,Z_{m}\right)}^{⊤}$. It then follows that cor$\left(X_{i},W_{j}\right)=\text{cor}\left(Z_{i},W_{j}\right)$. Now var(**Z**)=*k* **DΣD** and $\text{var}\left(W\right)=k{\left(D\Sigma D\right)}^{-1/2}D\Sigma D\times {\left(D\Sigma D\right)}^{-1/2}=kI.$ Also, $E\left[Z{\left\{W-E(W)\right\}}^{⊤}\right]=E\left[Z{\left\{{\left(D\Sigma D\right)}^{-1/2}Z\right\}}^{⊤}\right]=E\left(ZZ^{⊤}\right){\left(D\Sigma D\right)}^{-1/2}=$ $\text{var}\left(Z\right){\left(D\Sigma D\right)}^{-1/2}=k{\left(D\Sigma D\right)}^{1/2}$. Hence $cov(Z_{i},W_{j})$ is the (*i*,*j*) entry of $\left[k{\left(D\Sigma D\right)}^{1/2}\right]$. Since $\text{var}\left(Z_{i}\right)=\text{var}\left(W_{j}\right)=k$, both $\text{cor}(Z_{i},W_{j})$ and $\text{cor}(X_{i},W_{j})$ equal the (*i*,*j*)th entry of ${\left(D\Sigma D\right)}^{1/2}$. Similar reasoning shows that ${\text{cor}}_{s}\left(X_{i},{\hat{W}}_{j}\right)$ is the (*i*,*j*)th entry of ${\left(\hat{D}\hat{\Sigma }\hat{D}\right)}^{1/2}$.

Proof of Theorem 5 Part (i) follows from reasoning similar to the first steps of the proof of Theorem 1. To prove (ii), let $\Phi =\Gamma D\Sigma D\Gamma^{⊤}$ and let ***V***=***Y***−E(***Y***), so that E(***V***)=0 and var(**V**)∝**Φ**. Put $W^{*}=CV$, so that $\text{var}(W^{*})=I$ since $C^{⊤}C=\Phi^{-1}$. It then follows that $\Sigma_{i=1}^{m}\left[{\left\{\text{var}(Y_{i})\right\}}^{1/2}\text{cor}\left(Y_{i},W_{i}^{⋄}\right)\right]=\Sigma_{i=1}^{m}\left[{\left\{\text{var}(V_{i})\right\}}^{1/2}\text{cor}\left(V_{i},W_{i}^{*}\right)\right]=\Sigma_{i=1}^{m}cov\left(V_{i},W_{i}^{*}\right)=V^{⊤}W^{*}=V^{⊤}CV$. From Lemma 2, $V^{⊤}CV$ is maximised when $C=\Phi^{-1/2}={\left(\Gamma D\Sigma D\Gamma^{⊤}\right)}^{-1/2}$. Additionally, ${\left(\Gamma D\Sigma D\Gamma^{⊤}\right)}^{-1/2}$ is the unique symmetric square‐root of ${\left(\Gamma D\Sigma D\Gamma^{⊤}\right)}^{-1}=\Gamma {\left(D\Sigma D\right)}^{-1}\Gamma^{⊤}$. As $\left[\Gamma {\left(D\Sigma D\right)}^{-1/2}\Gamma^{⊤}\right]\left[\Gamma {\left(D\Sigma D\right)}^{-1/2}\Gamma^{⊤}\right]=\Gamma {\left(D\Sigma D\right)}^{-1}\Gamma^{⊤}$, it follows that ${\left(\Gamma D\Sigma D\Gamma^{⊤}\right)}^{-1/2}=\Gamma {\left(D\Sigma D\right)}^{-1/2}\Gamma^{⊤}$. Part (iii) follows immediately from (ii) and the definition of **Y**. The proof of (iv) is analogous to the proof of Theorem 3. Part (v) is immediate from equation (7).

## Appendix B Orthogonal matrices from contrasts

Suppose the first four *x*‐axes are rotated by the transformation ${\left(Y_{1},\ldots ,Y_{4}\right)}^{⊤}=\Gamma_{d}{\left(X_{1}^{⋄},\ldots ,X_{4}^{⋄}\right)}^{⊤}$, where

(24) $$\Gamma_{d}=\left(\begin{array}{cccc} 2^{-1} & 2^{-1} & -2^{-1} & -2^{-1}\\ 2^{-1/2} & -2^{-1/2} & 0 & 0\\ 0 & 0 & 2^{-1/2} & -2^{-1/2}\\ 2^{-1} & 2^{-1} & 2^{-1} & 2^{-1} \end{array}\right)$$

and $X_{j}^{⋄}=\left(X_{j}-\mu_{j}\right)/\sigma_{j}$ for *j*=1…,4, so that the $X_{j}^{⋄}$ are standardised *x*‐variables. It is readily checked that $\Gamma_{d}$ is an orthogonal matrix. The new variables are

$$\begin{array}{cc} & Y_{1}=\left(X_{1}^{⋄}+X_{2}^{⋄}\right)/2-\left(X_{3}^{⋄}+X_{4}^{⋄}\right)/2\;Y_{2}=\left(X_{1}^{⋄}-X_{2}^{⋄}\right)/2^{1/2}\\ & Y_{3}=\left(X_{3}^{⋄}-X_{4}^{⋄}\right)/2^{1/2}\;Y_{4}=\left(X_{1}^{⋄}+X_{2}^{⋄}+X_{3}^{⋄}+X_{4}^{⋄}\right)/2 \end{array}$$

Thus $Y_{1}$ is the difference between the average of the first two $X^{⋄}$ variables and the average of the other two $X^{⋄}$ variables, $Y_{2}$ and $Y_{3}$ are each proportional to the difference between a pair of $X^{⋄}$ variables, and $Y_{4}$ is proportional of the average of the four $X^{⋄}$ variables. Consequently $Y_{1},\ldots ,Y_{4}$ may all be meaningful combinations of the *x*‐variables, although this is obviously context dependent. The point of this example is that the top three rows of $\Gamma_{d}$ form a (non‐unique) set of orthogonal contrasts and contrasts can prove useful when meaningful linear combinations of variables are sought. It is certainly the case that in the analysis of experiments, contrasts among factor levels are commonly constructed for that reason.

More generally, one approach to finding rotation matrices that give meaningful new variables is to look for a set of meaningful orthonormal contrasts. Suppose the first *d* axes are to be rotated, so that linear combinations of $X_{1}^{⋄},\ldots ,X_{d}^{⋄}$ are to be formed. A complete set of orthonormal contrasts consists of *d*−1 linear combinations

(25) $$Y_{i}=\sum_{j=1}^{d}\alpha_{ij}X_{j}^{⋄}i=1,\ldots ,d-1,$$

such that $\Sigma_{j=1}^{d}\alpha_{ij}=0$, $\Sigma_{j=1}^{d}\alpha_{ij}^{2}=1$ and, for *i*≠*k*(*k*=1,…,*d*−1), $\Sigma_{j=1}^{d}\alpha_{ij}\alpha_{kj}=0$. The set of orthonormal contrasts is not unique, giving flexibility in constructing the $Y_{i}$. To form a rotation matrix from these contrasts, we set the (*i*,*j*)th entry of $\Gamma_{d}$ equal to $\alpha_{ij}$ (*i*=1,…,*d*−1;*j*=1,…,*d*) and the (*d*,*j*)th entry of $\Gamma_{d}$ equal to $d^{-1/2}$ (*j*=1,…,*d*).
